# Supercooling Agent Icilin Blocks a Warmth-Sensing Ion Channel TRPV3

**DOI:** 10.1100/2012/982725

**Published:** 2012-04-01

**Authors:** Muhammad Azhar Sherkheli, Guenter Gisselmann, Hanns Hatt

**Affiliations:** ^1^Department of Cell Physiology, Ruhr University Bochum, 44801 Bochum, Germany; ^2^Department of Pharmaceutical Sciences, Faculty of Pharmacy, Superior University, Raiwind Road, Lahore 54660, Pakistan

## Abstract

Transient receptor potential vanilloid subtype 3 (TRPV3) is a thermosensitive ion channel expressed in a variety of neural cells and in keratinocytes. It is activated by warmth (33–39°C), and its responsiveness is dramatically increased at nociceptive temperatures greater than 40°C. Monoterpenoids and 2-APB are chemical activators of TRPV3 channels. We found that Icilin, a known cooling substance and putative ligand of TRPM8, reversibly inhibits TRPV3 activity at nanomolar concentrations in expression systems like *Xenopus laeves* oocytes, HEK-293 cells, and in cultured human keratinocytes. Our data show that icilin's antagonistic effects for the warm-sensitive TRPV3 ion channel occurs at very low concentrations. Therefore, the cooling effect evoked by icilin may at least in part be due to TRPV3 inhibition in addition to TRPM8 potentiation. Blockade of TRPV3 activity by icilin at such low concentrations might have important implications for overall cooling sensations detected by keratinocytes and free nerve endings in skin. We hypothesize that blockage of TRPV3 might be a signal for cool-sensing systems (like TRPM8) to beat up the basal activity resulting in increased cold perception when warmth sensors (like TRPV3) are shut off.

## 1. Introduction

Thermosensation is believed to be directly mediated by sensory neurons of the dorsal root ganglia (DRGs) that terminate as free nerve endings within the dermal and epidermal layers of the mammalian skin [1–3]. Thermosensitive transient receptor potential (thermo-TRPs) ion channels are a subset of the transient receptor potential (TRP) super family of cation channels, which are believed to act as molecular sensors of temperature [4] because all six, when expressed in naive cells (human embryonic kidney cells, Chinese hamster ovary cells, *Xenopus* oocytes) have the amazing property of rendering cells temperature sensitive. In mammals temperature detection is assumed to be accomplished through concerted actions of thermo-TRPs, that is, TRPA1, TRPM8, and TRPV1-V4 each covering a defined threshold of temperature from below 17°C to 52°C [5, 6]. The expression of most of these thermo-TRPs in primary afferent neurons is consistent with a key role in thermal transduction at cellular levels. Transient receptor potential vanilloid-3 (TRPV3) is expressed in mammalian keratinocytes [7, 8] in addition to its expression in the epithelium of tongue and nose [9]. A TRPV3 null mouse shows impaired thermotaxis behavior over hot and innocuous temperature ranges with no other obvious sensory impairment [10]. Rodents, carrying constitutively active TRPV3 mutant, show hair loss and atopic dermatitis like lesions [11], and its activation inhibits hair growth in humans [12]. Increased TRPV3 expression is involved in breast tenderness in human females [13] and in traumatic tissue injury [14, 15]. In addition to temperature and metabolites of inflammatory pathway, natural products like terpenoids can activate or modulate TRPV3 functions [9, 16, 17]. All of these findings strongly indicate that TRPV3 plays a critical role in a variety of functions performed by mammalian skin. 

The prevailing model that temperature is directly sensed by cell bodies of DRG neurons [18] calls into question whether keratinocytes-expressed TRPV3 [10] channels are directly involved in thermosensation. This question becomes even more important when most of the members of thermo-TRPs channel subset are expressed in keratinocytes; for example, TRPM8 (which senses normal cooling or mild cold) and TRPV1, which are sensors for noxious heat (41°C and above). Whether these different members of TRP family hetero-multimerize in keratinocytes or not and what might be the likely implications of such hetero-multimerization, one perspective that can be explicitly stated is their simultaneous presence is highly likely to effect the kinetic behaviour of each individual member. For example, coexpression or simultaneous presence of TRPV1 is shown to block desensitization of TRPA1 [19] by blocking the apparent internalisation of TRPA1 channels. TRPV1 and TRPV3 are coexpressed in human DRGs and interact with each other in heterologous expression systems [20]. Whether such binding interactions play similar role in native tissue for TRPV3 and TRPV1 is not known.

Taking into account the proposed role of keratinocytes in the sensation of ambient temperature, the most important question that could be asked is: what exactly might be the role of a warmth sensor in proximity of a coldsensor when a stimulus impinges upon the sensory neuron containing cooling information? Or what might be the likely consequences for the basal activity of a coldsensor when its proximate-warmth-sensing partner is blocked? Alternatively, is it possible to enhance the cooling effect transduced by a cooling sensor just by blocking warmth sensor in addition to positively modulating basal activity of cold sensors such as TRPM8?

In the present study we tried to answer this question by studying the behaviour of TRPV3 as affected by icilin, a cooling-substance introduced by Wei and Seid [21] in 1983 but since then has been reported to activate TRPA1 [22–25] and TRPM8 [26], both of which are cold sensors in mammals. This investigation becomes even more interesting when the effects of menthol on TRP ion channels are bracketed in. Since TRPM8 is also a marker for prostate cancer [27], and cooling substances (e.g., menthol) are predicted to inhibit progression of certain human melanomas [28, 29], it becomes extremely interesting to analyze the interaction of Icilin, with other thermosensors like TRPV3. As menthol has been shown to be an agonist for TRPA1, TRPV3 and TRPM8 [17, 30], in the present investigation it was attempted to see whether icilin has any effect on TRPV3. It was found that icilin blocks TRPV3 in low micromolar concentration ranges. This leads to the suggestion that the prevention of TRPV3 activity might be an important trigger to “up-shoot” TRPM8 activity, leading to enhanced sense of cooling associated with icilin-like substances.

## 2. Methods

### 2.1. Expression Vectors for TRP Channels

The mouse TRPV3 (mTRPV3) cDNA was a generous gift from Dr. David Julius (UCSF, CA, USA). For efficient expression in *Xenopus* oocytes, cDNA was subcloned by a PCR-based standard method into the oocyte expression vector pSGEM [31].

### 2.2. Synthesis and Injection of TRP cRNA

The generation of cRNA was performed by standard methods as described elsewhere [23, 24, 32, 33]. In order to use plasmids containing cloned cDNA as a template for *in vitro* transcription, plasmids were linearized downstream of the end of the cDNA. Capped RNAs were synthesized in the presence of capping analogue *m* ^7^G(5′)ppp(5′)G using the AmpliCap-T7 MessageMaker Kit (Epicentre, Madison, WI, USA). RNA was ethanol-precipitated and redissolved in RNase-free water to give a final concentration of 1 *μ*g/*μ*L. Ovarian lobes were obtained from mature female *Xenopus laevis* anaesthetized by immersion in 0.15% 3-aminobenzoic acid ethyl ester. Ovarian tissue was removed and placed in Barth's solution (88 mM NaCl, 1 mM KCl, 0.82 mM MgSO_4_, 0.33 mM Ca(NO_3_)_2_, 0.41 mM CaCl_2_, 2.4 mM NaHCO_3_, 5 mM Tris-HCl, pH 7.4, 100 U/mL penicillin, 50 *μ*g/mL streptomycin). After treatment of the ovarian tissue with collagenase (type I, 4 mg/mL in Ca^2+^-free Barth's solution) for two hours at room temperature, the oocytes were incubated overnight in fresh Barth's solution (15°C). After 24 h, mature healthy oocytes (stages V to VI) were selected for cytoplasmic injection of cRNA (about 50 nl per oocyte; approximate cRNA concentration 1 *μ*g/*μ*L) with a sharp pipette using a pressure injector (npi PDES 04T, Tamm, Germany). Afterwards, injected oocytes were placed again in fresh Barth's solution and incubated at 16–18°C. Oocytes were tested for functional expression of TRP channels after 3 to 5 days.

### 2.3. Electrophysiological Recordings in Oocytes

Two-electrode voltage-clamp recording was used to obtain current responses to applied substances. Drugs were diluted to the final concentration in either Ca^2+^-containing (115 mM NaCl, 2.5 mM KCl, 1.8 mM CaCl_2_, 200 *μ*M flufenamic acid, 10 mM HEPES, pH 7.4) or Ca^2+^-free (115 mM NaCl, 2.5 mM KCl, 10 mM HEPES, 10 mM EGTA, pH 7.4) standard extracellular solution (SES) as indicated in the text. Agonists were applied by means of a multibarrel single-tip superfusion device or by manual application. Application time was usually 10–20 seconds. Electrodes were pulled from borosilicate glass using a Kopf vertical pipette puller and backfilled with 3 M KCl. Oocytes were constantly held at −40 mV using a command from the amplifier and evoked current signals were recorded with a two-electrode voltage-clamp amplifier (TURBO TEC-03, npi, Tamm, Germany) and acquired using the PCLAMP software (Axon Instruments, Sunnyvale, CA, USA).

### 2.4. Cell Culture and Transfection of HEK293 Cells

HEK293 cells were maintained under standard conditions in a minimum essential medium supplemented with 10% fetal bovine serum, 100 units/mL penicillin and streptomycin, and 2 mM L-glutamine. Semiconfluent cells were transfected in 35 mm dishes (Becton Dickinson, Heidelberg, Germany) using the Ca^2+^-phosphate-precipitation technique as described elsewhere [34]. Measurements were done 24–48 hr after transfection.

### 2.5. Electrophysiology in HEK293 Cells

Recordings were performed using the whole-cell mode of the patch-clamp technique. Cells were maintained in an extracellular recording solution containing (in mM) 140 NaCl, 5 KCl, 1 MgCl_2_, 10 HEPES, and pH 7.4. Patch electrodes were pulled from borosilicate glass (1.2 mm O.D.×1.17 mm I.D., Harvard apparatus, Edenbridge, Kent, UK) and fire polished to 4–6 MΩ tip resistance using a horizontal pipette puller (Zeitz Instr., Munich, Germany). The pipette solution contained (in mM) 140 KCl, 1 MgCl_2_, 0.1 CaCl_2_, 5 EGTA, 10 HEPES, pH 7.4 for recordings.

### 2.6. Single-Cell Calcium Imaging

Human primary keratinocytes (kindly provided by Dr. F. Jacobsen and Dr. L Steinstrsser; Klinik für Plastische Chirurgie und Schwerbrandverletzte, BG-kliniken Bergmannsheil, Bocum) were cultured in 3 : 1 DMEM : HamF-10 medium containing 10% FBS, 1% penicillin/streptomycin, 5 *μ*g/mL human insulin (Sigma), 1 *μ*M isoproterenol (Sigma), 24.3 *μ*g/mL adenine (Sigma), 08 g/mL hydrocartisone (Sigma), 20 ng/mL human epidermal growth factor (hEGF, Sigma), and 1.35 ng/mL 3,3′,5-triiodo-L-thyronine sodium salt (Sigma) at 37°C under 6% CO2 and 95% humidity conditions. Primary cell used no longer than at passage 5 were grown to a confluence of no more than 70% and splitted using Trypsin/EDTA. All cell culture components were obtained from GibCO/Invitrogen unless otherwise stated. 

Either HaCat cells or primary keratinocytes grown in 35 mm dishes were incubated for 30 min (HaCat) or 45 min (keratinocytes) at 37°C with loading buffer containing Ringer solution (pH 7.4) and 3 *μ*M fura-2-AM (Molecular Probes). Calcium imaging was performed using the multiway wavelength illumination system POLYCHROME II (T.I.L.L Photonics GmbH, Planegg, Germany) for excitation. Changes in cytosolic calcium concentrations were analyzed using a PCO interline chip camera. For acquisition and calculation of fluorescence signals obtained from excitation of the dye at 340 and 380 nm of the T.I.L.L vision software was used.

## 3. Statistics

All data were analysed for statistical significance using Sigma-Stat software (version 2.03) with *P values <0.05, **P < 0.005 and, ***P < 0.001 as significant differences in means. Data are expressed as mean ± S.E.M. of 6 to 73 independent measurements of responses under similar experimental conditions unless otherwise stated.

## 4. Results

### 4.1. Inhibition of TRPV3 Activity by Icilin in Oocytes

**Figure 1:**
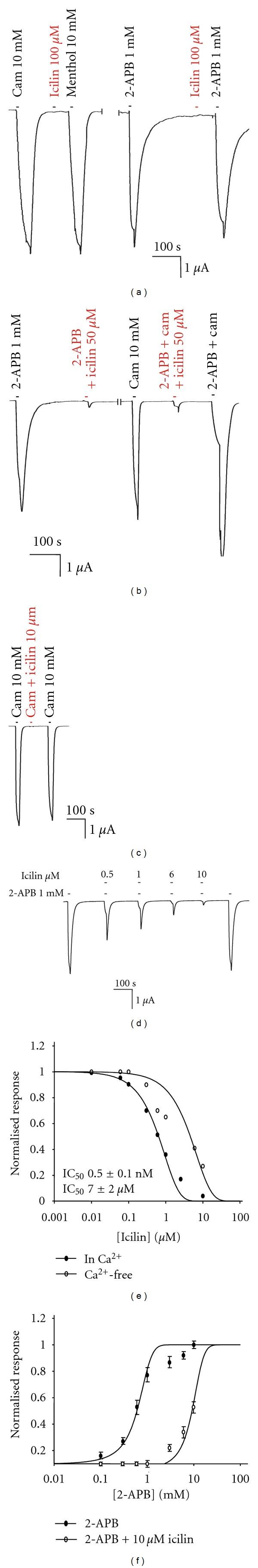
Block of mTRPV3 activity by icilin in oocytes. (a) A representative trace showing camphor, menthol, and 2-APB activation of mTRPV3, whereas icilin could not activate in Ca^2+^-containing SES. (b) A representative trace showing block of mTRPV3 activation by icilin in Ca^2+^-free SES. (c) Block of camphor-induced mTRPV3 currents by icilin. (d) Dose-dependent block of mTRPV3 by icilin in Ca^2+^-containing SES. (e) Inhibition curve for icilin's IC_50_ values. (f) Reversibility of inhibition of mTRPV3 by increasing 2-APB concentrations. (*n* = 6 in each case).

As menthol has been shown to be an agonist for mouse TRPV3 [17, 30], it was exciting to determine whether icilin, a supercooling substance and an agonist of TRPM8 has a similar effect on mTRPV3. mTRPV3 expressing oocytes were challenged with 1 mM 2-APB, 10 mM camphor and 100 *μ*M icilin. Camphor, and 2-APB exposure elicited macroscopic currents in TRPV3 expressing oocytes in Ca^2+^-containing extracellular solution, whereas icilin showed no activation (Figure 1(a)). After initial activation of mTRPV3, mixes of icilin and 2-APB or camphor were used to reproduce mTRPV3-mediated currents elicited either by camphor or 2-APB. Surprisingly, icilin blocked 2-APB and also a mix of 2-APB and camphor induced currents (Figure 1(b)). A similar protocol was used to determine whether camphor activation of mTRPV3 is also blocked by icilin. A complete block of camphor-induced currents in the presence of 10 *μ*M icilin was observed (Figure 1(c)). Higher concentrations of camphor could not be tested due to poor aqueous solubility of the substance.

The next attempt was to determine if this block is dose dependent. For such experiments different icilin concentrations (from 10 nM to 10 *μ*M) were used. While keeping the 2-APB concentration at 1 mM (EC_50_ 533 *μ*M [17]) increasing concentrations of icilin showed a gradual decrease in magnitude of 2-APB evoked currents. The 2-APB induced current was insignificant in the presence of 10 *μ*M icilin (Figure 1(d)). The IC_50_ value calculated for inhibition of mTRPV3 activity by icilin in Ca^2+^-containing SES was 0.5 ± 0.1 *μ*M and in Ca^2+^-free SES 7 ± 2 *μ*M (Figure 1(e)). The block of mTRPV3 by icilin could be overpowered by using higher 2-APB concentrations (3, 6, or 10 mM; Figure 1(f)) and was reversed after washout. The channel was responsive to repeated challenge of agonists after washout of icilin.

**Figure 2:**
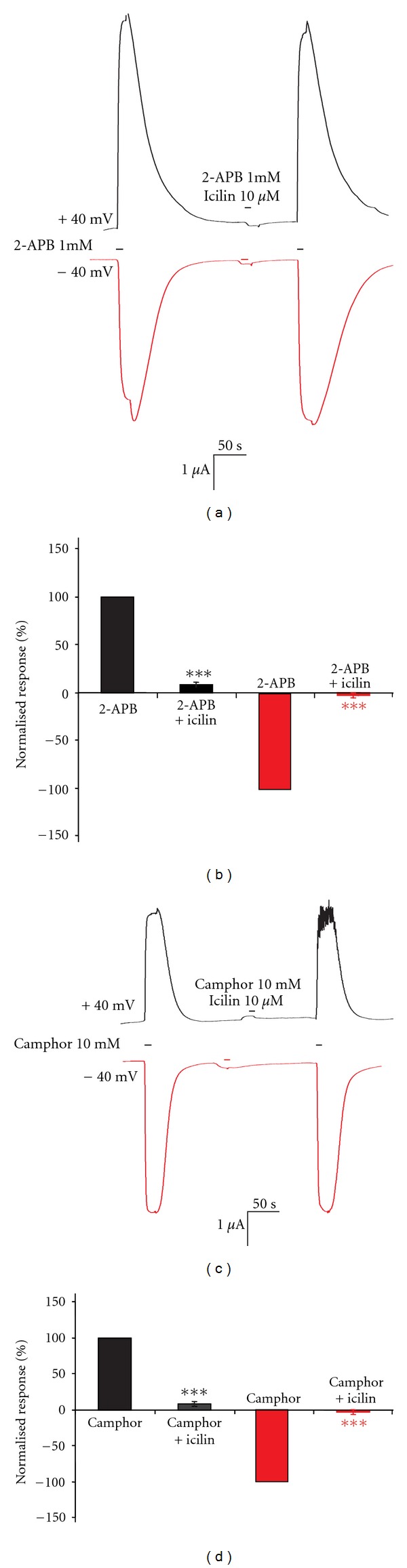
(a) Representative recordings showing an inhibition of mTRPV3 activity at different holding potentials. The upper and lower part of the trace shows a block of 2-APB-induced mTRPV3 activity at + or −40 mV holding potentials, respectively. (b) Quantification of the data from six experiments as explained above. (c) The upper and lower part of the trace shows a block of camphor induced mTRPV3 activity at + or −40 mV holding potentials, respectively. (d) Quantification of the data from six experiments as explained above. All values are expressed as mean ± SEM.

To check the influence of membrane potential on the block of mTRPV3 activity by icilin, two different holding potentials were employed to monitor the behavior of mTRPV3 and its response to 2-APB and camphor (in the presence or absence of icilin). Oocytes expressing mTRPV3 were held at +40 mV. Application of 1 mM 2-APB resulted in strong outward currents (8.41 ± 0.3 *μ*A *n* = 9). After a washout with SES the current decayed quickly displaying typical mTRPV3 property (Figure 2(a)). In the subsequent step, repeating the same procedure in the presence of 10 *μ*M icilin, an almost complete block (0.48 ± 0.08 *μ*A; ~94% decrease in current amplitude; = 9*P* < 0.00001) to 2-APB-induced currents was observed (Figures 2(a) and 2(b)). Similarly, 10 mM camphor evoked robust outward currents (4.19 ± 0.5 *μ*A *n* = 9) at +40 mV holding potentials, which decreased drastically (0.27 ± 0.1 *μ*A; ~93% decrease *n* = 9  *P* < 0.001) in the presence of 10 *μ*M icilin (Figures 2(c) and 2(d)). 

### 4.2. Inhibition of mTRPV3 Activity by Icilin in HEK293 Cells

**Figure 3:**
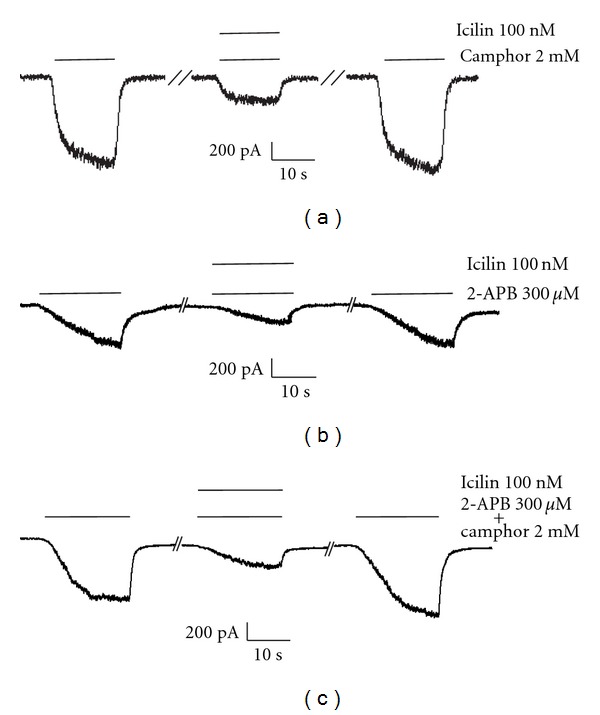
(a) A representative recording from patch-clamp experiment showing camphor-induced mTRPV3 activity inhibited by 100 nM icilin (61% inhibition; *n* = 6). HEK293 cells expressing mTRPV3 were held at –40 mV holding potential at ~33°C. (b) A representative recording from patch-clamp experiment showing 2-APB-induced mTRPV3 activity inhibited by 100 nM icilin (68% inhibition; *n* = 7) under similar conditions as above. (c) A representative recording from patch-clamp experiment showing icilin-dependent (100 nM) inhibition of mTRPV3 activity induced by both camphor and 2-APB (74% inhibition; *n* = 7) under similar conditions as above.

To test whether mTRPV3 blockage by icilin is reproducible in mammalian cells, mTRPV3 was expressed in HEK293 cells and investigated using whole-cell patch clamp. HEK293 cells expressing mTRPV3 were clamped at −40 mV constant potential. Expression was analyzed by an exposure of either 2-APB or camphor,respectively. Currents evoked by 300 *μ*M 2-APB could be blocked by 100 nM Icilin (Figure 3(a); 68% reduction in amplitude). Similarly, 10 mM camphor-induced currents were blocked ~61% in the presence of 100 nM icilin (Figure 3(b)). When a mixture of camphor and 2APB was applied a block of 75% was obtained (Figure 3(c)).

### 4.3. Inhibition of TRPV3 Activity by Icilin in HaCat Cells

**Figure 4:**
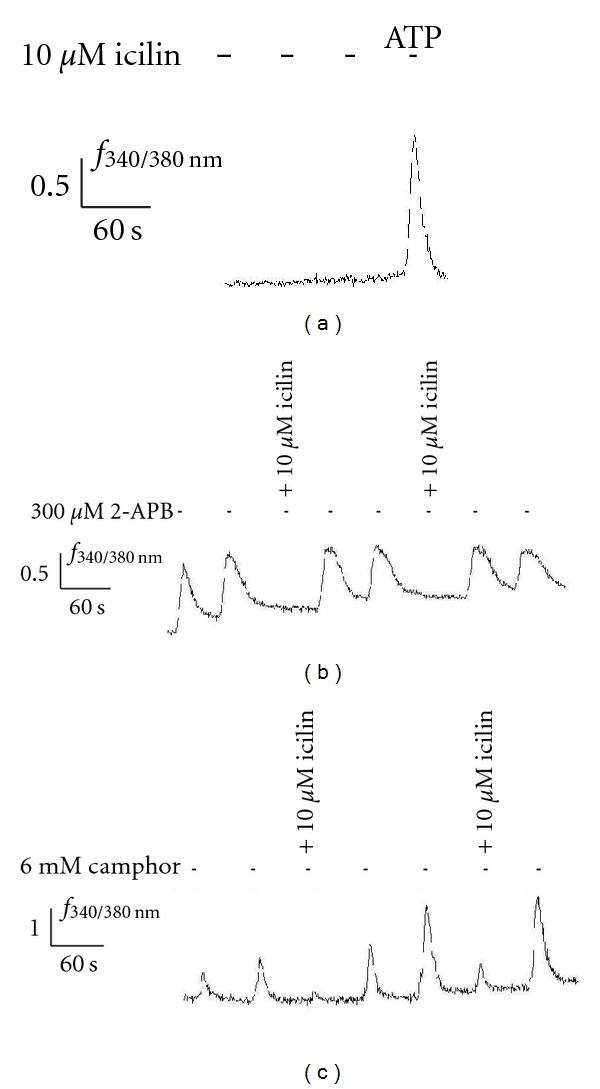
Block of TRPV3 in keratinocytes. Representative ratio-fluoremetric recordings of fura-2-loaded human primary keratinocytes are shown. Changes in cytosolic Ca^2+^ levels upon stimulation are depicted as fluorescence ratio (*f*_340_/*f*_380 *nm*_) and displayed as a function of time. Transient stimulation by either 300 *μ*M 2-APB (b) or 6 mM camphor (c) results in a Ca^2+^-influx. In contrast, stimulation by 10 *μ*M icilin had no effect on the intracellular calcium concentration (a). The physiological state of the cells was controlled by application of 100 *μ*M ATP at the end of the experiment. Upon coapplication of icilin induced Ca^2+^-signals by 2-APB (b) or camphor. (c) were dramatically reduced, respectively. Bars above denote the application points of the stimuli (black: stimulus; grey: coapplication of icilin).

As described earlier, mTRPV3 is predominantly expressed in keratinocytes [8]. We have reported previously [33] that stimulation with potent TRPV3 agonists like either 2-APB or camphor induces changes in intracellular calcium levels in the keratinocyte-derived HaCaT cell line. To demonstrate that icilin may have inhibitory effects on native TRPV3 channels, single cell calcium imaging experiments were performed on human primary keratinocytes-derived HaCat cells and keratinocytes. Initially, positive stimulation of the cells was monitored by 5 seconds application of either 300 *μ*M 2-APB (Figure 4(b)) or 6 mM camphor (Figure 4(c)). In both cases cells responded repeatedly to either substance. The amplitudes of successive Ca^2+^-signals did not vary significantly with 2-APB, whereas the camphor-induced Ca^2+^-signal reached its maximum amplitude at the second application. In contrast, icilin alone never induced activation of keratinocytes (Figure 4(a); *n* = 24) or HaCat cells (data not shown). Coapplication of icilin with either 2-APB (*n* = 73) or camphor (*n* = 66) resulted in a strong decrease of the signal either 100% or 80% of the cells, respectively. While 2-APB-induced responses were always reduced to base level, for camphor residual activity was observed in 50% of the cells (see Figures 4(b) and 4(c)). In addition, some variations in the blocking effect of icilin was noticed in keratinocytes obtained from different donors, for example, keratinocytes obtained from breast of females versus keratinocytes from circumcised forskin of male children. The detailed investigation about reasons for these variations are underway.

### 4.4. Block of Human TRPV3 in Oocytes

**Figure 5:**
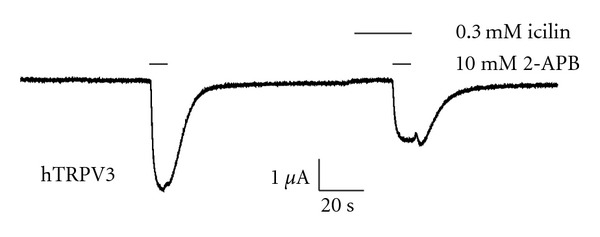
Original trace showing block of human TRPV3 by icilin. Since lower concentrations of icilin were not effective in case of hTRPV3 so higher doses were tested. 0.3 mM of icilin blocked ~50% of response induced by 10 mM 2-APB. The oocyte was preincubated in icilin before application of 2-APB. The experiment was carried out in Ca^2+^-free SES.

To check that if human TRPV3 (hTRPV3) is also blocked by icilin as is the case with mTRPV3. hTRPV3 expressed in oocytes was challenged with different concentrations of 2-APB in the presence and absence of 10 *μ*M icilin (a concentration which totally abolished mTRPV3 responses Figures 1(b) and 1(c)). Interestingly, this concentration of icilin was not much effective to block 2-APB-induced currents mediated by recombinant hTRPV3 (data not shown). However, when higher concentrations of icilin were employed, the block was obvious and significant as shown in Figure 5.

## 5. Discussion

TRPV3 is strongly expressed in the outermost layer of the skin (keratinocytes) in addition to its robust presence in the epithelium of the tongue and the nose [9, 35, 36]. It is profusely involved in thermal sensation at warm and hot temperature ranges. It has been suggested that layer of keratinocytes can be seen as a large and continuous sensory organ directly involved in the assessment of ambient temperatures [10]. Modulation of the function of thermo-TRP ion channels is believed to be the molecular basis for sensory properties of many natural compounds. For example, menthol is now known to produce its cooling effects by activating TRPM8 [37] and burning pain sensations by activation of TRPV3 [17, 30, 38]. Since TRPM8 is also a target of the cooling substance icilin, it was reasonably interesting to investigate whether icilin also activates TRPV3. In the present investigation it was found that as opposed to menthol icilin inhibits TRPV3 activity. This TRPV3 block might be an additional mechanism contributing to super-cooling sensations associated with icilin-like substances.

Icilin activates TRPM8 in the presence of extracellular calcium. In contrast, in the absence of calcium TRPM8 activation is virtually nonexisting [26, 39, 40]. Strong inhibition of TRPV3-mediated currents in *Xenopus* oocytes and HEK293 cells at low micromolar ranges of the drug was found; both in the presence and absence of extracellular calcium. In the absence of extracellular calcium, the inhibitory effect of icilin is significantly affected but not abolished (~50–90% inhibition; see Figures 1(b), 1(e), and 3). This is in contrast to its effects on TRPM8, where virtually no significant activation is observed in the absence of calcium. Thus, it appears that calcium seemingly enhances TRPV3 inhibition by icilin, whereas in case of TRPM8 calcium acts as a fundamental cofactor for the activation.

An important variation is noticeable with camphor activation of TRPV3 in human keratinocytes (Figure 4(c)). While 2-APB-elicited currents were usually abolished in all expression systems by icilin, camphor-evoked currents could not always be blocked in keratinocytes (*n* = 30; 30% of measured cells). This apparent anomaly might be because of bimodal kinetic behavior of TRPV3 [41]. The channels keep shifting between I_1_ and I_2_ modes. It may therefore display differential kinetics with two structurally different sets of chemical agonists. Since camphor binds to a different molecular locus as compared to 2-APB. This binding might be happening in such a way that it is capable of increasing open-probability state of the channels even in the presence of an inhibitor like icilin in at least 30% of the cells. This observation leads to the assumption that icilin might be a state-specific blocker for TRPV3. Potential heteromultimeric complexes of TRPV2/V3 and TRPV1/V3 [42, 43] should also be considered in cases where no inhibition to camphor-induced calcium influx was observed. Last but not least, camphor may be interacting with some other channels explaining its nonspecific action in some keratinocytes.

The inhibition of TRPV3 by icilin in oocytes and its reproducibility in HEK293 cells and human primary keratinocytes may have important implications for general understanding of sensory transduction mechanisms in peripheral neurons. These findings point towards a potential cooling mechanism. It is suggested that the cooling effect of icilin is not only produced by its activation of TRPM8 or TRPA1. The inhibition of warmth-sensing TRPV3 ion channels may at least contribute to the overall cooling efficacy. The thermal sensors apparently act in a concerted fashion. Thus, it may be safe to assume that blocking of warmth sensors is a “kind of signal” for the activation of cold sensors. The exact molecular dynamics of such an interaction in peripheral sensory neurons or keratinocytes are currently unknown, and thus, could be extremely exciting for future investigations. Last but not least, sensory illusions such as erroneous perception of warmth with cold stimuli (thermal grill illusion) might be the result of activation of warmth sensors like TRPV3 with cooling stimulus (like menthol).

## Abbreviations

2-APB: 2-aminoethoxy-di-phenyl borate
HEK-293: Human embryonic kidney 293
TRP: Transient receptor potential
DRG: Dorsal root ganglion
SES: Standard extra cellular solution.

## References

[1] Hensel H (1981). Neural processes in long-term thermal adaptation. *Federation Proceedings*.

[2] Patapoutian A (2003). TRP channels and somatic sensory receptors. *The FASEB Journal*.

[3] Takashima Y, Daniels RL, Knowlton W, Teng J, Liman ER, McKemy DD (2007). Diversity in the neural circuitry of cold sensing revealed by genetic axonal labeling of transient receptor potential melastatin 8 neurons. *Journal of Neuroscience*.

[4] Clapham DE (2003). TRP channels as cellular sensors. *Nature*.

[5] Jordt SE, McKemy DD, Julius D (2003). Lessons from peppers and peppermint: the molecular logic of thermosensation. *Current Opinion in Neurobiology*.

[6] Reid G (2005). ThermoTRP channels and cold sensing: what are they really up to?. *Pflugers Archiv*.

[7] Chung MK, Lee H, Mizuno A, Suzuki M, Caterina MJ (2004). TRPV3 and TRPV4 mediate warmth-evoked currents in primary mouse keratinocytes. *Journal of Biological Chemistry*.

[8] Peier AM, Reeve AJ, Andersson DA (2002). A heat-sensitive TRP channel expressed in keratinocytes. *Science*.

[9] Xu H, Delling M, Jun JC, Clapham DE (2006). Oregano, thyme and clove-derived flavors and skin sensitizers activate specific TRP channels. *Nature Neuroscience*.

[10] Moqrich A, Hwang SW, Earley TJ (2005). Impaired thermosensation in mice lacking TRPV3, a heat and camphor sensor in the skin. *Science*.

[11] Asakawa M, Yoshioka T, Matsutani T (2006). Association of a mutation in TRPV3 with defective hair growth in rodents. *Journal of Investigative Dermatology*.

[12] Borbíró I, Lisztes E, Tóth BI (2011). Activation of transient receptor potential vanilloid-3 inhibits human hair growth. *Journal of Investigative Dermatology*.

[13] Singh M, Gopinath R (2005). Topical analgesia for chest tube removal in cardiac patients. *Journal of Cardiothoracic and Vascular Anesthesia*.

[14] Frederick J, Buck ME, Matson DJ, Cortright DN (2007). Increased TRPA1, TRPM8, and TRPV2 expression in dorsal root ganglia by nerve injury. *Biochemical and Biophysical Research Communications*.

[15] Facer P, Casula MA, Smith GD (2007). Differential expression of the capsaicin receptor TRPV1 and related novel receptors TRPV3, TRPV4 and TRPM8 in normal human tissues and changes in traumatic and diabetic neuropathy. *BMC Neurology*.

[16] Hu HZ (2006). Potentiation of transient receptor potential vanilloid receptor 3 (TRPV3) sensing function by ethanol. *Gastroenterology*.

[17] Vogt-Eisele AK, Weber K, Sherkheli MA (2007). Monoterpenoid agonists of TRPV3. *British Journal of Pharmacology*.

[18] Cesare P, Mcnaughton P (1996). A novel heat-activated current in nociceptive neurons and its sensitization by bradykinin. *Proceedings of the National Academy of Sciences of the United States of America*.

[19] Akopian AN, Ruparel NB, Jeske NA, Hargreaves KM (2007). Transient receptor potential TRPA1 channel desensitization in sensory neurons is agonist dependent and regulated by TRPV1-directed internalization. *Journal of Physiology*.

[20] Smith GD, Gunthorpe MJ, Kelsell RE (2002). TRPV3 is a temperature-sensitive vanilloid receptor-like protein. *Nature*.

[21] Wei ET, Seid DA (1983). AG-3-5: a chemical producing sensations of cold. *Journal of Pharmacy and Pharmacology*.

[22] Sherkheli MA, Vogt-Eisele AK, Bura D, Beltrán Márques LR, Gisselmann G, Hatt H (2010). Characterization of selective trpm8 l igands and their structureactivity response (s.a.r) relationship. *Journal of Pharmacy and Pharmaceutical Sciences*.

[23] Sherkheli MA (2010). *Trip through the Pharmacology of Hot- and Cold Sensing TRP Ion Channels: A Hope for Better Painkillers and Anti-Inflammatory drugs*.

[24] Sherkheli MA (2008). Menthol derivative WS-12 selectively activates transient receptor potential melastatin-8 (TRPM8) ion channels. *Pakistan Journal of Pharmaceutical Sciences*.

[25] Story GM, Peier AM, Reeve AJ (2003). ANKTM1, a TRP-like channel expressed in nociceptive neurons, is activated by cold temperatures. *Cell*.

[26] McKemy DD, Neuhausser WM, Julius D (2002). Identification of a cold receptor reveals a general role for TRP channels in thermosensation. *Nature*.

[27] Tsavaler L, Shapero MH, Morkowski S, Laus R (2001). Trp-p8, a novel prostate-specific gene, is up-regulated in prostate cancer and other malignancies and shares high homology with transient receptor potential calcium channel proteins. *Cancer Research*.

[28] Li Q, Wang X, Yang Z, Wang B, Li S (2010). Menthol induces cell death via the TRPM8 channel in the human bladder cancer cell line T24. *Oncology*.

[29] Yamamura H, Ugawa S, Ueda T, Morita A, Shimada S (2008). TRPM8 activation suppresses cellular viability in human melanoma. *American Journal of Physiology*.

[30] Macpherson LJ, Hwang SW, Miyamoto T, Dubin AE, Patapoutian A, Story GM (2006). More than cool: promiscuous relationships of menthol and other sensory compounds. *Molecular and Cellular Neuroscience*.

[31] Villmann C, Bull L, Hollmann M (1997). Kainate binding proteins possess functional ion channel domains. *Journal of Neuroscience*.

[32] Sherkheli MA (2007). Selective TRPM8 agonists: a novel group of neurophathic analgesics. *FEBS Journal*.

[33] Sherkheli MA, Benecke H, Doerner JF (2009). Monoterpenoids induce agonist-specific desensitization of transient receptor potential vanilloid-3 (TRPV3) ion channels. *Journal of Pharmacy and Pharmaceutical Sciences*.

[34] Zufall F, Hatt H, Firestein S (1993). Rapid application and removal of second messengers to cyclic nucleotide- gated channels from olfactory epithelium. *Proceedings of the National Academy of Sciences of the United States of America*.

[35] Chung MK, Lee H, Caterina MJ (2003). Warm temperatures activate TRPV4 in mouse 308 keratinocytes. *Journal of Biological Chemistry*.

[36] Ahmed MK, Takumida K, Ishibashi T, Hamamoto T, Hirakawa K (2009). Expression of transient receptor potential vanilloid (TRPV) families 1, 2, 3 and 4 in the mouse olfactory epithelium. *Rhinology*.

[37] Mccoy DD, Knowlton WM, Mckemy DD (2011). Scraping through the ice: uncovering the role of TRPM8 in cold transduction. *American Journal of Physiology*.

[38] Vay L, Gu C, McNaughton PA The thermo-TRP ion channel family: properties and therapeutic implications.

[39] Chuang HH, Neuhausser WM, Julius D (2004). The super-cooling agent icilin reveals a mechanism of coincidence detection by a temperature-sensitive TRP channel. *Neuron*.

[40] Andersson DA, Chase HWN, Bevan S (2004). TRPM8 activation by menthol, icilin, and cold is differentially modulated by intracellular pH. *Journal of Neuroscience*.

[41] Chung MK, Güler AD, Caterina MJ (2005). Biphasic currents evoked by chemical or thermal activation of the heat-gated ion channel, TRPV3. *Journal of Biological Chemistry*.

[42] Hellwig N, Albrecht N, Harteneck C, Schultz G, Schaefer M (2005). Homo- and heteromeric assembly of TRPV channel subunits. *Journal of Cell Science*.

[43] Gunthorpe MJ, Benham CD, Randall A, Davis JB (2002). The diversity in the vanilloid (TRPV) receptor family of ion channels. *Trends in Pharmacological Sciences*.

